# Similarities and differences between systemic juvenile idiopathic arthritis and adult-onset Still’s disease: a multicenter Spanish study

**DOI:** 10.1007/s00296-024-05658-6

**Published:** 2024-09-23

**Authors:** Jordi Antón, Juan Manuel Mosquera, Joan Calzada, Estíbaliz Iglesias, Andrea Zacarías, Alejandro Olivé, Violeta Bittermann, Tania Rodríguez Lorenzo, Agustín Remesal, Cristian Quintana-Ortega, Laura Nuño-Nuño, Angel Robles-Marhuenda, Jaime de Inocencio, María Martín-López, Patricia E. Carreira, Anahy M. Brandy-García, Susana Holgado, Marisol Camacho-Lovillo, Alberto Ruiz-Román, Daniel Clemente, Javier Narváez, José Campos, Judith Sánchez-Manubens, Pilar Bernabéu, Jenaro Graña, Carmen Vargas, Vera Ortiz-Santamaria, Santos Castañeda, María Jesús García de Yébenes, Loreto Carmona

**Affiliations:** 1 Hospital Sant Joan de Déu, Barcelona, Spain; 2 Department of Surgery and Medical-Surgical Specialties, Obstetrics, Gynecology and Pediatrics. Medicine and Health Sciences School, Universitat de Barcelona, Barcelona, Spain; 3 Hospital Universitari Germans Trias i Pujol, Badalona, Spain; 4 Centro de Salud de Añaza, Gerencia de Atención Primaria del Área de Salud de Tenerife, Tenerife, Spain; 5 Hospital Universitario La Paz, Madrid, Spain; 6 Hospital Universitario 12 de Octubre, Madrid, Spain; 7 Hospital Universitario Virgen del Rocío, Seville, Spain; 8 Hospital Niño Jesús, Madrid, Spain; 9 Hospital Universitari Bellvitge, Barcelona, Spain; 10 Hospital Universitario Puerta de Hierro Majadahonda, Madrid, Spain; 11 Hospital Universitari Parc Taulí, Barcelona, Spain; 12 Hospital General Universitario Dr Balmis, ISABIAL, Alicante, Spain; 13 Complejo Hospitalario Universitario de A Coruña, A Coruña, Spain; 14 Hospital Universitario Virgen Macarena, Seville, Spain; 15 Hospital de Granollers, Granollers, Spain; 16 Hospital Universitario de la Princesa, IIS-Princesa, Madrid, Spain; 17 Instituto de Salud Musculoesquelética, Madrid, Spain

**Keywords:** Juvenile arthritis, Adult-onset still’s disease, Macrophage activation syndrome, Epidemiology

## Abstract

To describe the characteristics of systemic juvenile idiopathic arthritis (sJIA) and adult-onset Still’s disease (AOSD), compare their presentation and evolution, and analyse possible complication predictors. Multicenter study. Data were retrieved from a hospital-based study of patients with a diagnosis or suspected diagnosis of sJIA or AOSD according to the responsible physician and followed-up for at least one year. Descriptive variables (classification criteria, clinical manifestations, complications, family, and personal history) were collected at disease onset and during follow-up. We present the clinical characteristics of 326 patients, 67% of whom had a diagnosis of sJIA and 33% of AOSD. Clinical manifestation frequencies were similar between the two groups, except for odynophagia, which was significantly more frequent in AOSD than in sJIA (78.4% vs. 25.5%; *p* < 0.0001). Among the complications, macrophage activation syndrome (MAS) was significantly more common in sJIA than in AOSD (24.4% vs. 9.5%; *p* = 0.002), to the extent that an sJIA diagnosis significantly increased the risk of MAS, together with serositis presence, and the need for biological therapy. Patients with sJIA and AOSD showed similar characteristics, supporting the idea that they are both part of Still’s disease, but are expressed at different ages. Differences in manifestations and complications might be due to different management between diseases and immune response maturity.

**Supplementary Information:**

The online version contains supplementary material available at 10.1007/s00296-024-05658-6.

## Introduction

The systemic onset form of chronic childhood arthritis was first described by George Frederic Still in 1897 [1]. Today, systemic juvenile idiopathic arthritis (sJIA) is defined by the *International League of Associations for Rheumatology* (ILAR) as arthritis occurring in one or more joints accompanied or preceded by fever, lasting at least two weeks, documented as daily for at least three days in a row, and presenting with one or more of the following signs and symptoms: erythematous evanescent rash (non-fixed), generalised lymph node enlargement, hepatomegaly or splenomegaly, and serositis [2]. In 1971, Bywaters published the first series of 14 adults with the same symptoms as those seen in the paediatric age group, thus defining the adult-onset Still’s disease (AOSD) concept [3]. Since then, different criteria have been proposed for the diagnosis of Still’s disease in this later age group, such as those of Calabro [4], Cush [5], Yamaguchi [6], and Fautrel [7]. Both diseases are now considered part of Still’s disease, and attempts are being made to establish common diagnostic and classification criteria.

Systemic JIA and AOSD are thought to be complex multigenic autoinflammatory diseases [8]. They primarily affect the innate immune system and are characterised by inappropriate phagocyte activation (neutrophils and macrophages) and a reduced role for adaptive immunity. Their aetiology is unknown, although some genetic factors and pathophysiological pathways are known, such as the involvement of the interleukins IL-1β, IL-18, and IL-6 [9].

Both are characterised by acute and recurrent inflammatory episodes resulting from a dysregulation in the control of the inflammatory process and present different forms of evolution—monocyclic, polycyclic, and persistent—with 25 to 50% of the patients displaying a chronic course [8, 10–14]. Systemic symptoms, i.e., fever, increased erythrocyte sedimentation rate (ESR) and C-reactive protein (CRP), polyarticular or hip involvement, radiological damage, need for glucocorticoids, increased immunoglobulin A (IgA), thrombocytopenia, and elevated serum ferritin levels (very important if glycosylated ferritin < 20% according to Fautrel) have all been described as poor prognostic signs, and major complications include amyloidosis and macrophage activation syndrome (MAS) [8, 15–17]. Regardless of age, Still’s disease poses multiple challenges due to its heterogeneity, the lack of specific clinical and biological features, the need for differential diagnosis, the lack of prognostic markers, as well as the disability risk, the impaired quality of life, and the occurrence of serious complications despite treatment [18].

Disease registries, defined by the grouping and follow-up of patients sharing the same diagnosis, are very useful for describing the natural history of a condition, analysing the time to a specific event, evaluating the effectiveness and safety of therapies, as well as having subgroups of patients with particular characteristics, generating new research hypotheses, and improving disease clinical management [19].

The peculiar characteristics of sJIA and AOSD call for a better understanding of these processes. For example, using different classification criteria makes it difficult to compare case series or cohorts. In addition, there are no standardised therapeutic protocols in adults. In this sense, creating an sJIA-AOSD registry would improve knowledge of this disease [20].

The objective of this work was to describe the characteristics of a sJIA-AOSD Spanish study, to compare their presentation and course, and to analyse the possible predictive factors for the development of complications.

## Methods

### Design

Multicentre study of prevalent cases.

### Population and recruitment

The target population were patients with sJIA or AOSD (prevalent and incident) seen in the paediatrics, rheumatology, or internal medicine departments of tertiary hospitals throughout Spain. Eligible patients (selection criteria) were those with suspected or confirmed diagnosis of sJIA or AOSD, regardless of the criteria used, and a minimum follow-up of 1 year in participating hospitals.

To increase the external validity of the sample, a minimum participation of 10 centres was established, distributed throughout the different counties, and the recruitment period was 10 months. Patients for whom it was impossible to complete 50% of the data from clinical records were excluded.

### Variables

Descriptive and predictive variables were collected and grouped into the following sections: (A) *epidemiological data*: onset forms and evolution, age and gender, educational level, place of residence, family and personal history (vaccinations, comorbidity, and obstetric history); (B) *diagnostic criteria* (ILAR, Cush, Yamaguchi and Fautrel); (C) *clinical variables of onset and evolution*, quality of life and functional capacity, laboratory parameters, and imaging tests; and (D) *complications* (damage measured by the Juvenile Arthritis Damage Index [JADI], MAS, amyloidosis confirmed by histology, and uveitis).

A form and a codebook were designed with information on the variables and their validation rules to guide the researchers in collecting information and to facilitate variable location and interpretation during the analysis phase. Data were collected on a specific platform designed ad hoc and hosted in an online application that guaranteed the confidentiality and security of the data included under current legislation on data protection and allowed for regular uploading of the information to a global database.

### Statistical analysis

The statistical analysis was carried out in different steps. First, the sample was described using measures of central tendency (mean and median) and dispersion (standard deviation and interquartile range) for quantitative variables, and absolute and relative frequencies for qualitative variables. Second, the two patient groups, adults and children, were compared using parametric or non-parametric hypothesis tests. To avoid the problem of multiple comparisons, summary variables were created for family history [autoinflammatory (Familiar mediterranean fever, Syndrome TRAPS, HIDS, CAPS, PAPA, Granulomatous arthritis, Still disease, Recurrent fever) and immunological processes (Lupus, Psoriasis, Inflammatory bowel disease, Spondyloarthropathy, Uveitis, Rheumatoid arthritis, Multiple Sclerosis, Diabetes)], comorbidity (infections, cardiovascular events, osteoporosis, lung diseases, cancer), and baseline characteristics.

In a third step, bi- and multivariate logistic regression models were used to analyse the possible predictive role of baseline clinical characteristics on the subsequent development of complications. The dependent variables in these models were the most frequent complications, and baseline clinical characteristics were entered as independent variables. Variables with clinical significance or bivariate p-value < 0.25 were included in the multivariate models. From a saturated model, successive models were run with stepwise elimination of variables with the lowest contribution. The final model was chosen as the most parsimonious (with fewer explanatory variables), clinically meaningful, and with the lowest Alaike and Bayesian information criteria (AIC and BIC). Separate models were used for each complication.

The data are available upon reasonable request.

## Results

The sample consisted of 326 patients (67% with sJIA and 33% with AOSD), belonging to 14 Spanish centres. Compared with sJIA patients, those with AOSD were more frequently women (67% vs. 53%; *p* = 0.020), had a longer time between symptom onset and diagnosis (median 0.13 vs. 0.08 years; *p* = 0.015), and had a higher frequency of family history (30% vs. 11%; *p* = 0.001) and comorbidity (51% vs. 30%; *p* = 0.001). The most frequently met diagnostic criteria were those of Yamaguchi (80%) without differences between diseases, except for the Fautrel criteria, which were significantly more common in sJIA patients (68% vs. 54%; *p* = 0.019).

Regarding clinical features, the main differences were the higher frequency of rash in sJIA patients, and of serositis, morning stiffness, arthralgia, odynophagia, and lymphadenopathy in AOSD patients. Persistent forms were the most common (39.5%), followed by monocyclic (37.0%), with a slight difference between both diseases (*p* = 0.042). Monocyclic form proportion increased with the year of diagnosis (14.3% in the 1980–1990 decade to 44.3% in 2011–2021; *p* < 0.0001). In terms of treatment needs, more sJIA patients required glucocorticoids (85% vs. 64%; *p* < 0.0001), without differences for biologics. Finally, the JADI index showed a higher joint damage score in sJIA patients than in AOSD patients (2.5 ± 7.2 vs. 0.8 ± 5.4; *p* = 0.009), that was reflected in the radiological damage. Virtually, all patients had ESR values ≥ 30 mm/h (93%), and 48% had ferritin levels above 1500ng/mL, without differences between age groups. Significant differences were found in other laboratory data. In AOSD patients, haemoglobin values < 12 g/dL and elevated transaminases above 40IU/L were more frequent than in sJIA patients. In contrast, more sJIA patients displayed platelet levels of 400,000/mm^3^ or higher. Most patients had normal chest X-rays (81%), echocardiograms (76%), and abdominal ultrasounds (62%), with no differences by age group. Of the 150 patients who underwent joint radiography, 31 (21%) had joint erosions, and 28 (18%) showed joint destruction (Table 1).

**Table 1:** Comparisons of the main clinical characteristics between the two groups

Variable	Total (*n* = 326)	Systemic JIA (*n* = 219)	AOSD (*n* = 107)	*p*-value
**Baseline characteristics**				
Fever (*n* = 311)	310 (99.7%)	212 (99.5%)	98 (100%)	1.000
Arthralgia (*n* = 316)	295 (93.3%)	194 (90.6%)	101 (99.0%)	0.003
Rash (*n* = 310)	278 (89.7%)	197 (92.9%)	81 (82.6%)	0.006
Hepatosplenomegaly (*n* = 306)	105 (34.3%)	68 (32.2%)	37 (38.9%)	0.252
Serositis (*n* = 304)	71 (23.4%)	41 (19.4%)	30 (32.3%)	0.015
Morning stiffness (*n* = 299)	167 (55.8%)	107 (51.9%)	60 (64.5%)	0.043
Constitutional syndrome (*n* = 309)	116 (37.5%)	72 (34.3%)	44 (44.4%)	0.085
Headache (*n* = 300)	38 (12.7%)	25 (12.0%)	13 (14.1%)	0.612
Odynophagia (*n* = 314)	134 (42.7%)	54 (25.5%)	80 (78.4%)	< 0.0001
Lymphadenopathy (*n* = 309)	107 (34.6%)	64 (30.5%)	43 (43.4%)	0.025
Symmetric arthritis (*n* = 252)	148 (58.7%)	101 (62.7%)	47 (51.6%)	0.086
Persistent arthritis (*n* = 231)	114 (49.3%)	80 (52.6%)	34 (43.0%)	0.167
**Evolution descriptors**				
Evolution type (*n* = 324)				0.042
• Monocyclic • Polycyclic • Persistent	120 (37.0%) 76 (23.5%) 128 (39.5%)	91 (41.7%) 47 (21.6%) 80 (36.7%)	29 (27.4%) 29 (27.4%) 48 (45.3%)	
Need for glucocorticoids (*n* = 299)	233 (77.9%)	170 (85.0%)	63 (63.6%)	< 0.0001
Need for biologics (*n* = 300)	196 (65.3%)	139 (68.5%)	57 (58.8%)	0.098
JADI: articular (*n* = 322) (mean ± SD)	1.9 ± 6.7	2.5 ± 7.2	0.8 ± 5.4	0.009
JADI: extra-articular (*n* = 302)	0.69 ± 1.25	0.73 ± 1.43	0.60 ± 0.77	0.101
**Laboratory parameters**				
ESR ≥ 30 (*n* = 302)	280 (92.7%)	190 (92.7%)	90 (92.8%)	0.975
Ferritin ≥ 1,500ng/mL (*n* = 269)	130 (48.3%)	78 (44.1%)	52 (56.5%)	0.052
Haemoglobin < 12 g/dL (*n* = 312)	68 (21.8%)	29 (14.0%)	39 (37.1%)	< 0.0001
Platelets ≥ 400,000/mm^3^ (*n* = 291)	155 (53.3%)	129 (63.5%)	26 (29.5%)	< 0.0001
Leukocytes ≥ 15,000/mm^3^ (*n* = 304)	141 (46.4%)	96 (47.8%)	45 (43.7%)	0.500
ALT ≥ 40U/L (*n* = 275)	109 (39.6%)	57 (30.8%)	52 (57.8%)	< 0.0001
AST ≥ 40U/L (*n* = 269)	125 (46.5%)	75 (41.0%)	50 (58.1%)	0.009
GGT ≥ 40U/L (*n* = 203)	122 (60.1%)	61 (50.8%)	61 (73.5%)	0.001
Cholesterol ≥ 200 mg/dL (*n* = 211)	59 (28.0%)	36 (26.3%)	23 (31.1%)	0.458
**Imaging tests**				
Chest X-ray: normal (*n* = 280)	226 (80.7%)	151 (82.1%)	75 (78.1%)	0.428
Echocardiogram: normal (*n* = 181)	137 (76.1%)	104 (79.4%)	33 (67.3%)	0.092
Abdominal US: normal (*n* = 220)	135 (62.2%)	98 (64.0%)	37 (57.8%)	0.387
Erosions (*n* = 243)	31	24 (25.8%)	7 (12.3%)	0.047
Destruction (*n* = 243)	28	22 (23.6%)	6 (10.5%)	0.045

Data represent n (%), except where other statistics are specified

Abbreviations: JIA, juvenile idiopathic arthritis; AOSD, adult-onset Still’s disease; US, ultrasound; JADI, Juvenile Arthritis Damage Index; ESR, erythrocyte sedimentation rate, ALT, alanine transaminase; AST, aspartate aminotransferase; GGT, gamma-glutamyltransferase, SD, standard deviation

The most common complications were chronic damage (radiologic or functional damage) (18%) and MAS (Ravelli criteria or pathology changes) (19%), which were significantly more common in sJIA patients than in AOSD patients (24% vs. 9%; *p* = 0.002). Less common complications were functional impairment (6%), impaired growth in sJIA patients (15%), cataracts (6%); osteoporotic fracture (5%), and depression (4%) (Table 2).

**Table 2:** Comparisons of complications (total and by age group)

Complications	Total	sJIA	AOSD	*p*-value
Chronic damage (*n* = 319)	59 (18.5%)	44 (20.7%)	15 (14.1%)	0.159
Functional impairment (*n* = 294)	18 (6.1%)	14 (7.2%)	4 (4.0%)	0.318
Amyloidosis (*n* = 317)	1 (0.3%)	-	1 (1.0%)	0.328
Stunting (*n* = 312)	32 (10.3%)	31 (14.6%)	1 (1.0%)*	< 0.0001
Avascular necrosis (*n* = 316)	5 (1.6%)	4 (1.9%)	1 (1.0%)	1.000
Cataracts (*n* = 316)	20 (6.3%)	15 (7.1%)	5 (4.8%)	0.624
Uveitis (*n* = 315)	1 (0.3%)	1 (0.5%)	-	1.000
Osteoporotic fracture (*n* = 314)	17 (5.4%)	12 (5.7%)	5 (4.9%)	1.000
Depression (*n* = 316)	13 (4.1%)	6 (2.8%)	7 (6.7%)	0.101
Other (*n* = 291)	28 (9.6%)	19 (10.1%)	9 (8.7%)	0.705
MAS: suspected/confirmed (*n* = 314)	61 (19.4%)	51 (24.4%)	10 (9.5%)	0.002

*The patient with AOSD and impaired growth had an age at onset of 18.2 years

Abbreviations: MAS, macrophage activation syndrome; sJIA, systemic juvenile idiopathic arthritis; AOSD, adult-onset Still’s disease

**Fig. 1:**
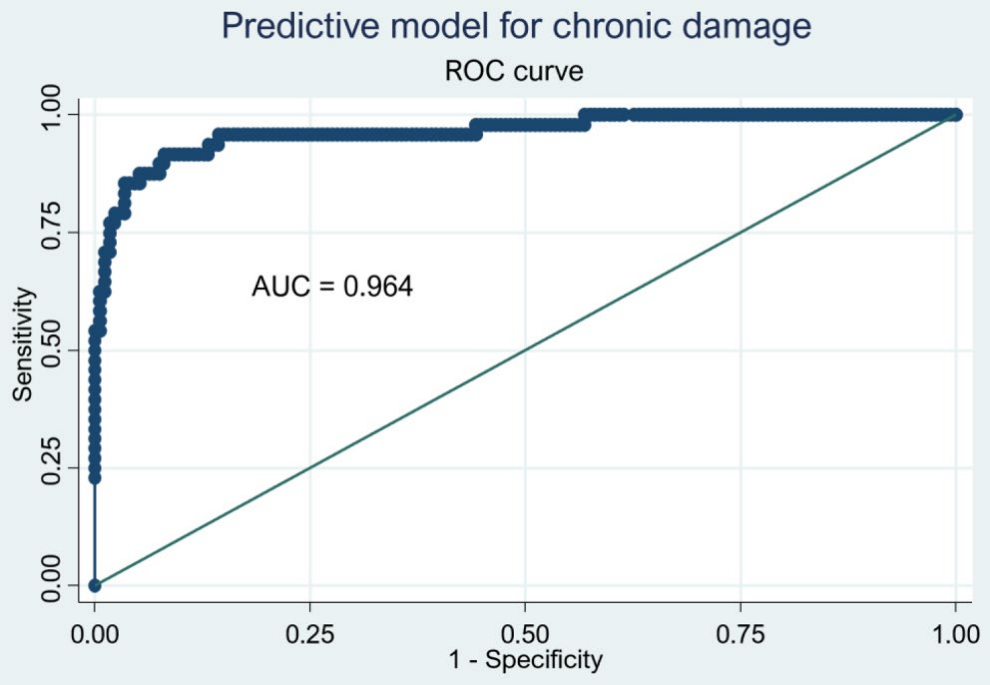
Chronic damage: model discrimination. ROC curve

Possible chronic damage predictors in bivariate analysis were a longer time since symptom onset (odds ratio [OR] = 1.15; *p* < 0.0001), morning stiffness (OR = 4.26; *p* < 0.0001), symmetrical (OR = 6.09; *p* < 0.0001) and persistent arthritis (OR = 4.04; *p* < 0.0001), number of swollen joints (OR = 1.16; *p* < 0.0001), higher score in joint (OR = 5.89; *p* < 0.0001) and extra-articular damage domains (OR = 2.67; *p* < 0.0001) of the JADI index, biological therapy (OR = 2.56; *p* = 0.009), and polycyclic (OR = 14.3; *p* = 0.001) or persistent (OR = 29.3; *p* < 0.0001) course. In the multivariate analysis, the only clinical characteristics associated with a higher chronic damage probability were a more extended time from symptom onset to the last visit (OR = 1.14; *p* < 0.0001), extra-articular damage in the JADI (OR = 5.49; *p* < 0.0001), and need for biological therapy (OR = 11.4; *p* < 0.0001) (Table 3). The discriminatory power of this model was very high, with an area under the ROC (Receiver Operating Characteristic) curve of 0.964 (Fig. 1).

**Table 3:** Baseline predictors of chronic damage

Determinant	Bivariate OR [95% CI] (*p*-value)	Multivariate OR [95% CI] (*p*-value)
Disease		
• AOSD • sJIA	1 1.58 [0.83–2.99] (0.161)	
Female gender	0.62 [0.35–1.10] (0.090)	
Family history	0.79 [0.28–2.17] (0.643)	
Years between symptom onset and diagnosis	1.19 [0.98–1.44] (0.082)	
Years between symptom onset and last visit	1.15 [1.10–1.21] (< 0.0001)	1.14 [1.07–1.22] (< 0.0001)
Baseline variables		
Rash	1.58 [0.53–4.71] (0.411)	
Morning stiffness	4.26 [2.05–8.87] (< 0.0001)	
Serositis	0.90 [0.44–1.82] (0.771)	
Hepatomegaly	0.94 [0.48–1.84] (0.857)	
Splenomegaly	1.07 [0.54–2.09] (0.850)	
Arthralgia	2.18 [0.49–9.65] (0.303)	
Constitutional syndrome	0.71 [0.38–1.33] (0.281)	
Odynophagia	0.76 [0.42–1.39] (0.380)	
Lymphadenopathy	0.40 [0.20–0.82] (0.012)	
Persistent arthritis	4.04 [2.01–8.13] (< 0.0001)	
Symmetrical arthritis	6.09 [2.62–14.20] (< 0.0001)	
No. swollen joints	1.16 [1.10–1.20] (< 0.0001)	
Comorbidity	0.90 [0.47–1.73] (0.759)	
JADI: joint damage	5.89 [3.26–10.64] (< 0.0001)	5.49 [2.78–10.83] (< 0.0001)
JADI: extra-articular damage	2.67 [1.97–3.61] (< 0.0001)	
Need for glucocorticoids	1.75 [0.78–3.92] (0.176)	
Need for biologics	2.56 [1.26–5.20] (0.009)	11.4 [1.71–76.2] (< 0.0001)
Type of evolution		
• Monocyclic • Polycyclic • Persistent	1 14.3 [3.16–64.4] (0.001) 29.3 [6.91–124.6] (< 0.0001)	
AUC (ROC curve)		0.967

Abbreviations: sJIA, systemic juvenile idiopathic arthritis; AOSD, adult-onset Still’s disease; US, ultrasound; JADI, Juvenile Arthritis Damage Index; AUC, area under the curve; OR, odds ratio; CI, confidence interval, ROC, Receiver Operating Characteristic

**Fig. 2:**
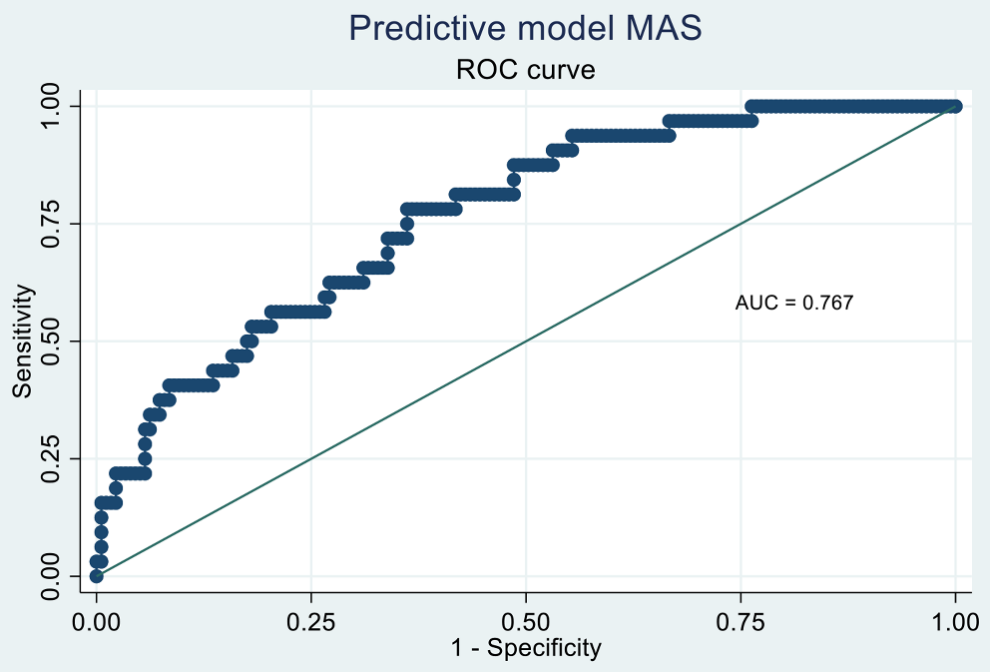
Macrophage activation syndrome (MAS): model discrimination. ROC curve

The baseline characteristics increasing MAS likelihood in the bivariate analysis were the clinical form of sJIA (OR = 3.31; *p* = 0.005), serositis (OR = 3.55; *p* < 0.0001), hepatomegaly (OR = 2.02; *p* = 0.037), splenomegaly (OR = 2.31; *p* = 0.014), need for biological therapy (OR = 4.74; *p* < 0.002), and persistent course (OR = 2.24; *p* = 0.040). In the multivariate model, the predictors associated with MAS development were the clinical form of sJIA (OR = 5.43; *p* = 0.013), serositis presence (OR = 4.55; *p* = 0.001), and need for biological therapy (OR = 4.37; *p* = 0.011). On the other hand, MAS probability decreased with time elapsed between symptom onset and the last visit, although this association did not reach statistical significance (Table 4). The discriminatory power of this model was moderate, with an area under the ROC curve of 0.767 (Fig. 2).

**Table 4:** Baseline predictors of macrophage activation syndrome (MAS)

Determinant	Bivariate OR [95% CI] (*p*-value)	Multivariate OR [95% CI] (*p*-value)
Disease		
• AOSD • sJIA	1 3.31 [1.43–7.68] (0.005)	1 5.43 [1.43–20.6] (0.013)
Female gender	0.71 [0.38–1.32] (0.278)	
Family history	1.47 [0.58–3.68] (0.415)	
Years between symptom onset and diagnosis	0.81 [0.47–1.40] (0.459)	
Years between symptom onset and last visit	0.94 [0.89–1.00] (0.061)	0.94 [0.88–1.00] (0.068)
Baseline variables		
Rash	1.27 [0.42–3.81] (0.670)	
Morning stiffness	0.99 [0.52–1.91] (0.990)	
Serositis	3.55 [1.83–6.90] (< 0.0001)	4.55 [1.88–11.01] (0.001)
Hepatomegaly	2.02 [1.04–3.92] (0.037)	
Splenomegaly	2.31 [1.19–4.50] (0.014)	
Arthralgia	1.60 [0.36–7.14] (0.539)	
Constitutional syndrome	1.29 [0.68–2.47] (0.437)	
Odynophagia	0.71 [0.37–1.39] (0.321)	
Lymphadenopathy	1.40 [0.73–2.70] (0.313)	
Persistent arthritis	0.81 [0.38–1.73] (0.580)	
Symmetrical arthritis	0.85 [0.42–1.74] (0.664)	
No. joints inflamed	0.99 [0.95–1.03] (0.650)	
Comorbidity	1.01 [0.50–2.03] (0.973)	
JADI: joint damage	1.00 [0.95–1.05] (0.999)	
JADI: extra-articular damage	1.15 [0.93–1.42] (0.184)	
Need for glucocorticoids	-	
Need for biologics	4.74 [1.80–12.5] (0.002)	4.37 [1.39–13.7] (0.011)
Type of evolution		
• Monocyclic • Polycyclic • Persistent	1 1.97 [0.83–4.66] (0.123) 2.24 [1.04–4.83] (0.040)	
AUC (ROC curve)		0.767

Abbreviations: sJIA, systemic juvenile idiopathic arthritis; AOSD, adult-onset Still’s disease; US, ultrasound; JADI, Juvenile Arthritis Damage Index; AUC, area under the curve; OR, odds ration; CI: confidence interval; ROC, Receiver Operating Characteristic

## Discussion

This study presented the characteristics of a Spanish sJIA-AOSD multicentre study and the analysis of complication predictors. The higher prevalence in females was similar to that published in other studies [9, 21, 22] and the age at diagnosis in sJIA (5.3 years) was similar to that of the CARRA group (*Childhood Arthritis and Rheumatology Research Alliance*) (4.7 years) [23] or the Pay series from Turkey (6 years) [21]. The onset age in AOSD patients (40.6 years) was similar to that of the Italian retrospective multicentre study (38.8 years) [24] and higher than that of the Turkish series (27 years) [21].

The shorter time between symptom onset and diagnosis (0.96 months in sJIA and 1.56 months in AOSD patients) compared with other series (1.56 months in sJIA and 3 months in AOSD patients) [21] may be related to different access to specialised units, or to better training of primary care teams and swift referral.

Clinical manifestation frequencies were similar between both groups, except for odynophagia, which was significantly more common in AOSD than in sJIA (78.4% vs. 25.5%). This difference has been also observed in other studies. For example, in Jamilloux’s comparison of previous series, only 15% of sJIA patients had odynophagia compared with 70% of those with AOSD [9]. Although this result may be related to the inclusion of odynophagia in Yamaguchi’s diagnostic criteria [6], it may also be due to younger patients with sJIA not reporting this symptom unless specifically asked to do so. In this regard, some studies conducted in adolescents with sJIA showed an odynophagia incidence similar to that observed in AOSD [25]. Serositis was also more frequent in AOSD (32.3% vs. 19.4%), although the difference was not significant, similar to that observed in the Italian registry [25]. The higher comorbidity frequency (51% vs. 30%; *p* = 0.001) in AOSD may be related to the older age of these patients.

There are currently no common diagnostic criteria for both presentation forms. Yamaguchi’s criteria were the most frequently met in our data (78.1% in sJIA and 85.0% in AOSD), as observed in other studies [26, 27]. Regarding prognosis, monocyclic forms were less prevalent in AOSD patients than in other series (41.7% in sJIA and 27.4% in AOSD) [28, 29]. The variability between the different series may be related to the retrospective nature of the studies, the emergence of new treatments, or changes in the disease course with early treatment initiation [30]. The higher proportion of monocyclic forms at later diagnosis dates could be explained by the greater number of patients treated with biological treatments in later decades.

Regarding complications, MAS was more common in sJIA patients than in AOSD patients (24.4% vs. 9.5%; *p* = 0.002), contrary to what was observed in the Italian and French series [9, 22]. Amyloidosis and articular lesions were less common than in older series, such as Cabane’s study published in 1990, probably due to current better diagnosis and access to new treatments [31]. The differences between sJIA and AOSD patients in articular JADI, but not in the extra-articular component, can be explained by the fact that joint involvement during growth in paediatric patients increases the damage risk.

The strengths of the present study are based on the availability of a large number of Still’s disease patients under real-world conditions, allowing to study the natural history of this rare disease [32, 33] and develop priority lines of research, such as common diagnostic criteria, prognostic markers, and analysis of later evolutionary phases, all of them from a multidisciplinary perspective (paediatric and adult rheumatologists). In this sense, this sample can serve as a basis for the creation of collaborative networks between different specialists.

However, the study is not exempt from limitations common to other registries [19], which relate to the retrospective nature and quality of the data collected [34], lack of representativeness of the sample, possible selection biases due to non-uniform assessment criteria, and incomplete patient follow-up [33]. There is currently an international project for developing and implementing an International *Autoinflammatory Disease Alliance* registry for patients with Still’s disease that attempts to overcome some of these problems [35].

In conclusion, patients with sJIA and AOSD showed similar characteristics, supporting the idea that they are two extremes within the spectrum of the same disease, expressed at different ages. A delay in diagnosis and a higher incidence of symptoms such as serositis and odynophagia stood out in AOSD. Among the complications, more significant joint damage has been observed in sJIA patients, and baseline factors associated with a more chronic damage presence have been identified. It is crucial to reach a consensus between paediatric and adult specialists to develop common classification criteria. This will allow prospective studies to include the full age spectrum of the disease and facilitate the development of treatment protocols.

## Electronic supplementary material

Below is the link to the electronic supplementary material.

Supplementary Material 1

## Appendix. The JIA-Still SERPE register components

Agustín Remesal, Hospital Universitario La Paz, Madrid.

Alejandro Olivé, Hospital Germans Trias y Pujol, Barcelona.

Alina Botenau, Hospital Ramón y Cajal, Madrid.

Ángel Robles Marhuenda, Hospital Universitario La Paz, Madrid.

Beatriz Bravo, Hospital Virgen de las Nieves, Granada.

Borja de Miguel Campo, Hospital Universitario 12 de Octubre, Madrid.

Carmen Vargas Lebron, Hospital Virgen Macarena, Sevilla.

Daniel Clemente Garulo, Hospital Niño Jesús, Madrid.

Esmeralda Nuñez, Hospital Materno Infantil de Málaga, Málaga.

Gisela Díaz-Cordovés Rego, Hospital Universitario Carlos Haya, Málaga.

Jaime de Inocencio, Hospital Universitario 12 de Octubre, Madrid.

Javier Narváez, Hospital Universitario de Bellvitge, Barcelona.

Jenaro Graña Gil, Hospital Universitario A Coruña, Coruña.

Jordi Antón López, Hospital Sant Joan de Deu, Barcelona.

Jordi Fiter, Hospital Son Espases, Palma de Mallorca.

Jose Alfredo Gómez-Puerta, Hospital Clinic, Barcelona.

Jose Campos Esteban, Hospital Universitario Puerta de Hierro Majadahonda, Madrid.

Judith Sánchez Manubens, Hospital Universitario Parc Taulí, Barcelona.

Julián Fernández Martín, Hospital Álvaro Cunqueiro, Vigo.

Laura Casas, HUN Sra de Candelaria, Tenerife.

Laura Nuño, Hospital Universitario La Paz, Madrid.

Mª del Pilar Hernández Jiménez, Hospital Universitario 12 de Octubre, Madrid.

Mª Sagrario Bustabad Reyes, Hospital Universitario de Canarias, Tenerife.

Mª Soledad Camacho Lovillo, Hospital Virgen del Rocío, Sevilla.

María del Carmen Pinedo, Hospital Universitario de Cruces, Bilbao.

María Teresa Silva Díaz, Hospital Universitario de La Coruña, Coruña.

Marta Medrano San Ildefonso, Hospital Universitario Miguel Servet, Zaragoza.

Patricia Carreira, Hospital Universitario 12 de Octubre, Madrid.

Santos Castañeda, Hospital La Princesa, Madrid.

Vera Ortiz Santamaría, Hospital General de Granollers, Barcelona.
