# Supplementary material for: Similarities and differences between systemic juvenile idiopathic arthritis and adult-onset Still’s disease: a multicenter Spanish study
Source: Rheumatol Int. 2024 Sep 23;44(12):2911–20. doi: 10.1007/s00296-024-05658-6 (PMC11618169; doi:10.1007/s00296-024-05658-6)
Supplement: Supplementary file 1 — Supplementary Material 1 [file 296_2024_5658_MOESM1_ESM.pdf]

Dr. Pau Ferrer Salvans
Secretario del CEIC Fundació Sant Joan de Déu

CERTIFICA

Que el CEIC Fundació Sant Joan de Déu en su reunión del día 06/04/2017, ha evaluado la propuesta del promotor referida al estudio:

Título: *"Registro Español de Artritis Idiopática Juvenil Sistémica (AIJS) y Enfermedad de Still del Adulto"*

Código Promotor: De AIJS a Still
Código Interno: EPA-05-17
Promotor: Sociedad Española de Reumatología

Protocolo	Versión 2 - 01/02/2017
Hoja Información de Paciente - Adulto	Versión 1 - 22/03/2017
Asentimiento - Adulto	Versión 1 - 22/03/2017
Hoja Información de Paciente - Representante legal menor	Versión 1 - 22/03/2017
Asentimiento - Representante legal menor	Versión 1 - 22/03/2017

1º. Considera que:

- El proyecto se plantea siguiendo los requisitos de la Orden SAS/3470/2009 del 16 de Diciembre de 2009 y las normas que lo desarrollan y su realización es pertinente.
- Se cumplen los requisitos necesarios de idoneidad del protocolo en relación con los objetivos del estudio y están justificados los riesgos y molestias previsibles para el sujeto, teniendo en cuenta los beneficios esperados.
- El procedimiento para obtener el consentimiento informado, incluyendo la hoja de información para los sujetos y el plan de reclutamiento de sujetos previstos son adecuados.
- La capacidad del investigador y sus colaboradores y las instalaciones y medios disponibles son apropiados para llevar a cabo el estudio.
- El alcance de las compensaciones económicas previstas no interfiere con el respeto a los postulados éticos.

2º. El CEIC Fundació Sant Joan de Déu, tanto en su composición, como en los PNT cumple con las normas de BPC (CPMP/ICH/135/95).

3º. La composición actual del CEIC de la Fundació Sant Joan de Déu es la siguiente:

Presidente:
- Jesús Pineda Sánchez

Edifici Docent Sant Joan de Déu - c. Santa Rosa, 39-57, 3a planta, 08950 Esplugues de Llobregat / Barcelona
Telèfon +34 93 600 97 51 - Fax +34 93 600 97 71 e-mail: info@fsjd.org web: www.fsjd.org

Secretario:

- Pau Ferrer Salvans

Vocales:

- Fernando Aguiló Martínez
- Clara Chamorro Pérez
- Ofelia Cruz Martínez (Hospital - HSJD - Hospital Sant Joan de Déu - Esplugues HSJD.1)
- Ángel del Campo Escota
- Beatriz Del Pino Gaya
- Rosa María Dueñas Herrero (Parc Sanitari Sant Joan de Déu)
- Sabel Gabaldon Fraile
- Maria Teresa Giner Muñoz (Hospital Sant Joan de Déu - Esplugues HSJD.1)
- Encarna Gómez Gamboa (Hospital Sant Joan de Déu - Esplugues HSJD)
- Iolanda Jordán García (Hospital Sant Joan de Déu - Esplugues HSJD)
- Joan Vinent Genestar (Hospital Sant Joan de Déu - Esplugues HSJD)
- Bernabé Robles Del Olmo (Parc Sanitari Sant Joan de Déu)
- María Eugènia Rey Abella

4º. Por lo que este CEIC emite un **INFORME FAVORABLE**.

5º. Este CEIC acepta que dicho estudio sea realizado en los siguientes CEIC/Centros por los Investigadores:

- HOSPITAL SANT JOAN DE DEU. Jordi Antón López.

Lo que firmo en Esplugues de Llobregat, a 27 de abril de 2017

Fdo:

Fundació Sant Joan de Déu
Comitè Ètic d'Investigació Clínic

Dr. Pau Ferrer Salvans
Secretario del CEIC Fundació Sant Joan de Déu

Edifici Docent Sant Joan de Déu - c. Santa Rosa, 39-57, 3a planta, 08950 Esplugues de Llobregat / Barcelona
Telèfon + 34 93 600 97 51 - Fax + 34 93 600 97 71 e-mail: info@fsjd.org web: www.fsjd.org
